# Supplementary material for: Tensin Regulates Fundamental Biological Processes by Interacting with Integrins of Tonsil-Derived Mesenchymal Stem Cells
Source: Cells. 2022 Jul 29;11(15):2333. doi: 10.3390/cells11152333 (PMC9367440; doi:10.3390/cells11152333)
Supplement: Supplementary file 1 [file cells-11-02333-s001.zip › Figure S1.pptx]

## Slide 1
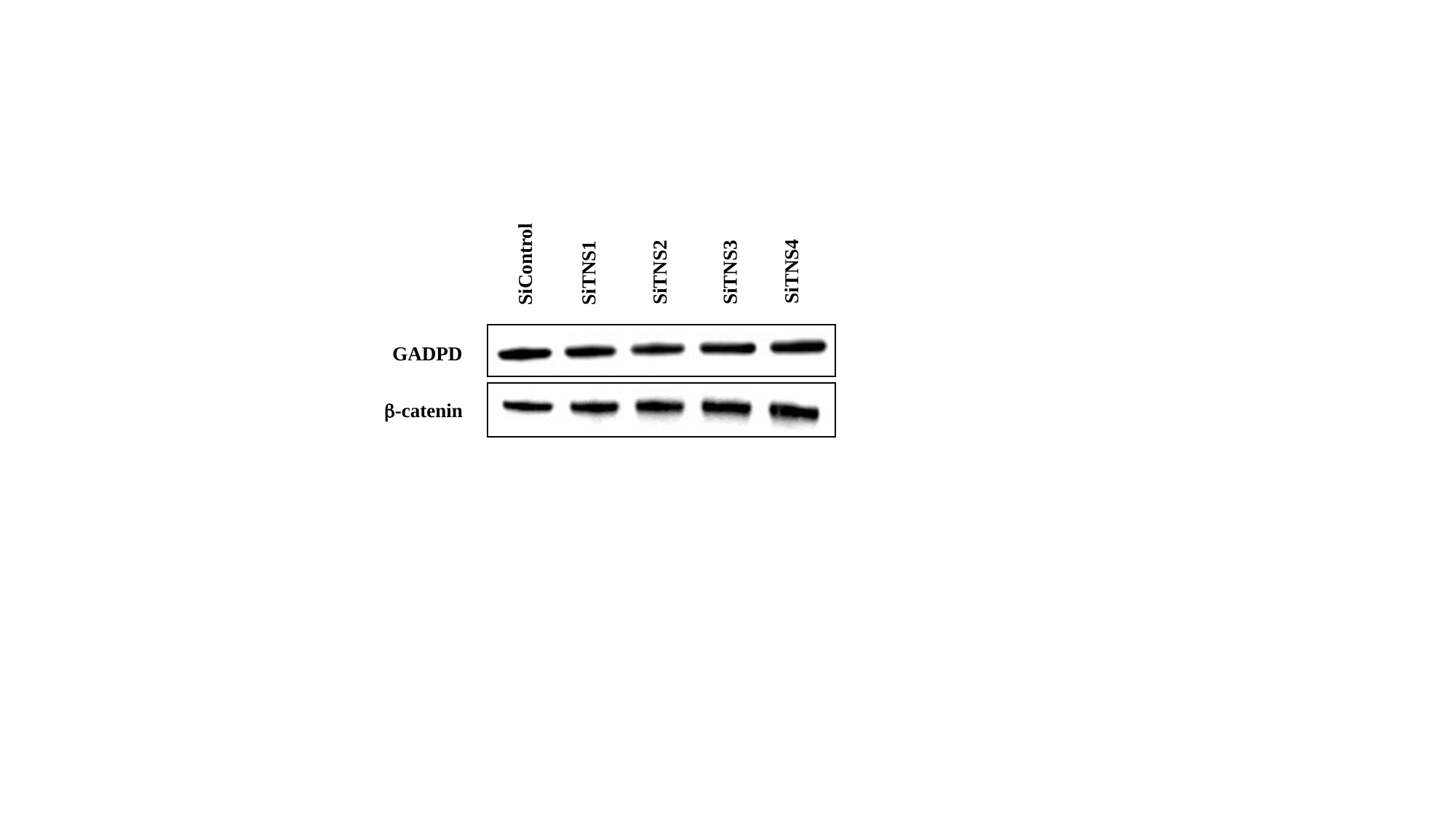

SiControl
SiTNS4
SiTNS3
SiTNS2
SiTNS1
GADPD
β-catenin

## Slide 2
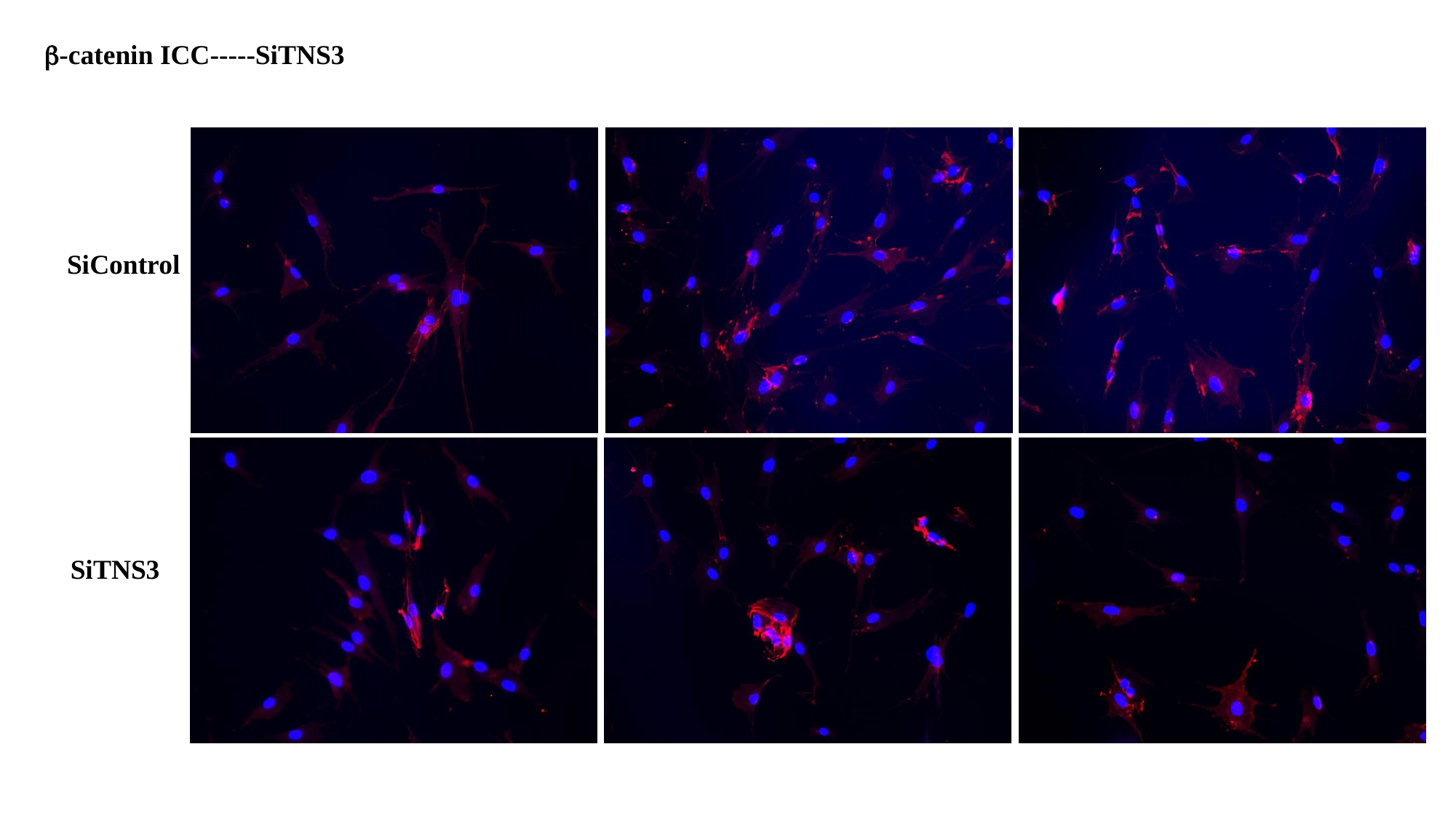

β-catenin ICC-----SiTNS3
SiControl
SiTNS3

## Slide 3
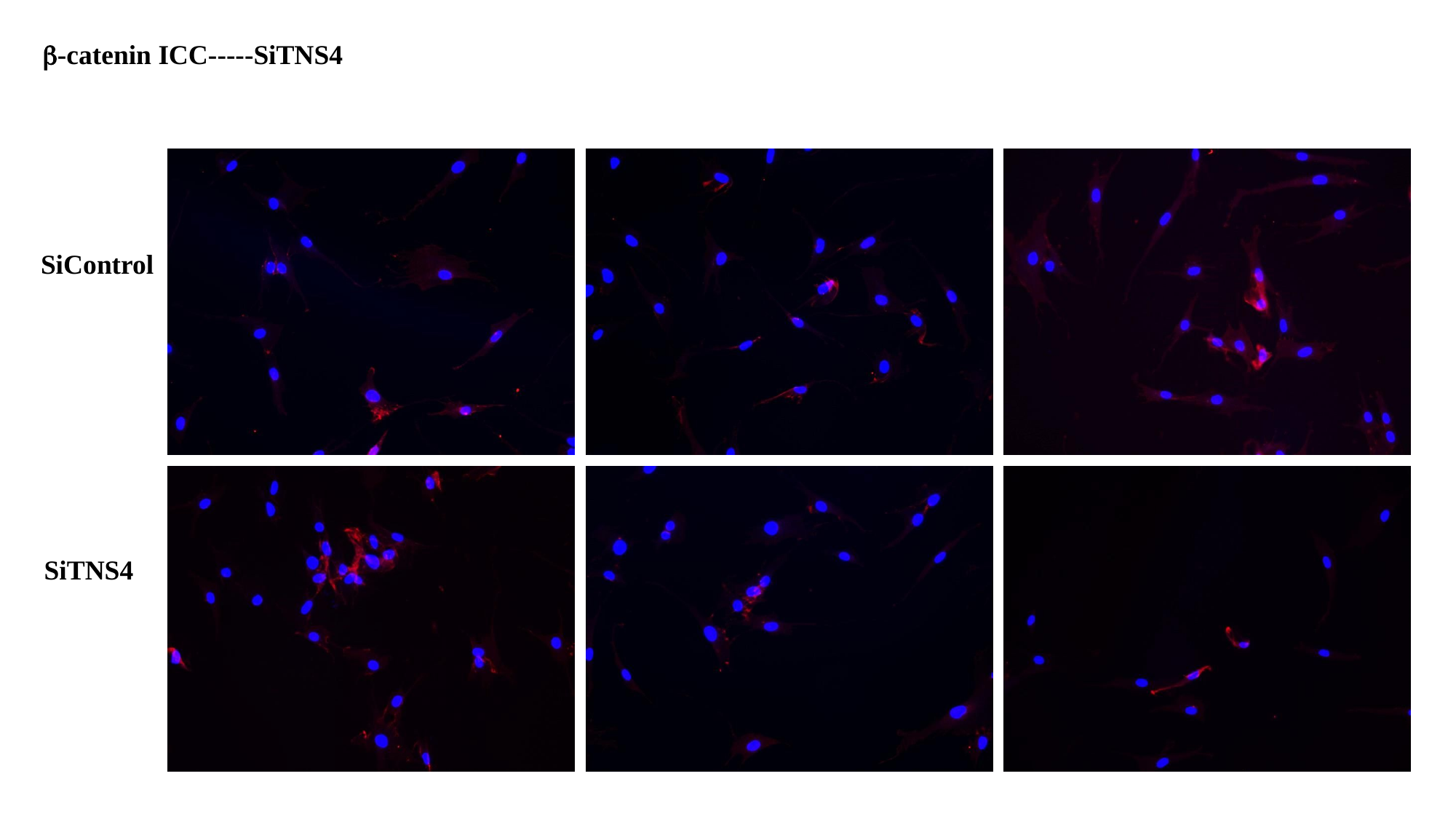

β-catenin ICC-----SiTNS4
SiControl
SiTNS4
