# Supplementary material for: Tensin Regulates Fundamental Biological Processes by Interacting with Integrins of Tonsil-Derived Mesenchymal Stem Cells
Source: Cells. 2022 Jul 29;11(15):2333. doi: 10.3390/cells11152333 (PMC9367440; doi:10.3390/cells11152333)
Supplement: Supplementary file 1 [file cells-11-02333-s001.zip › Figure S2.pptx]

## Slide 1
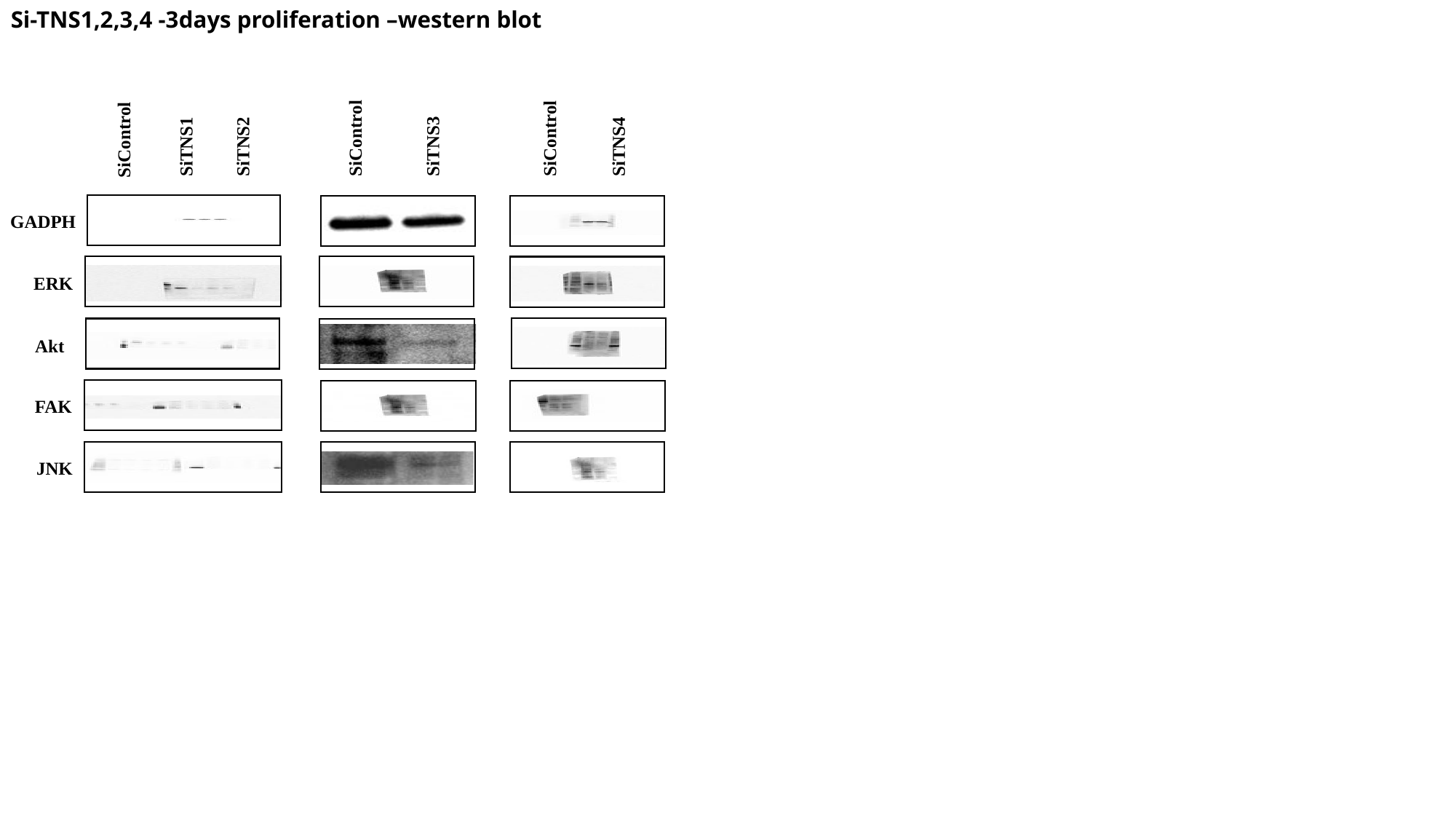

Si-TNS1,2,3,4 -3days proliferation –western blot
SiControl
SiControl
SiControl
SiTNS3
SiTNS2
SiTNS4
SiTNS1
GADPH
ERK
Akt
FAK
JNK
