# Supplementary material for: Tensin Regulates Fundamental Biological Processes by Interacting with Integrins of Tonsil-Derived Mesenchymal Stem Cells
Source: Cells. 2022 Jul 29;11(15):2333. doi: 10.3390/cells11152333 (PMC9367440; doi:10.3390/cells11152333)
Supplement: Supplementary file 1 [file cells-11-02333-s001.zip › Table S1.pdf]

Supplementary Table S1. Primer sequence used in real-time polymerase chain reaction (PCR).

Gene	Primer sequences
Sox-2	Forward 5′-GGGAAATGGGAGGGGTGCAAAAGAGG-3′
	Reverse 5′-TTGCGTGAGTGTGGATGGGATTGGTG-3′
Nanog	Forward 5′-AGTCCCAAAGGCAAACAACCCACTTC-3′
	Reverse 5′-TGCTGGAGGCTGAGGTATTTCTGTCTC-3′
Oct-4	Forward 5′-GACAGGGGGAGGGGAGGAGCTAGG-3′
	Reverse 5′-CTTCCCTCCAACCAGTTGCCCCAAAC-3′
C-myc	Forward 5′-AAAGGCCCCCAAGGTAGTTA-3′
	Reverse 5′-GCACAAGAGTTCCGTAGCTG-3′
p16	Forward 5′-GGGGAGAGTAGATAGCGGGC-3′
	Reverse 5′-AACCAATCAACCGAAAATTCCATA-3′
p19	Forward 5′-CTCTGCTCCCTGATAGCCCT-3′
	Reverse 5′-TGCGAAGGATTTTGAAGCGG-3′
p21	Forward 5′-GTCACTGTCTTGTACCCTTGTG-3′
	Reverse 5′-CGGCGTTTGGAGTGGTAGAAA-3′
CDC25	Forward 5′-CTTCCTTTACCGTCTGTC-3′
	Reverse 5′-AAACCATTCGGAGTGCTA-3′
Cyclin E	Forward 5′-GGATGTTGACTGCCTTAG-3′
	Reverse 5′-CACCACTGATACCCTGAAA-3′
Osteocalcin	Forward 5′- TCTATGACCTGCAGAGGGCT-3′
	Reverse 5′- ATAGCTCGTCACAAGCAGGG-3′
ALP	Forward 5′- TCCCCGCAACAGATCTCCTA-3′
	Reverse 5′- AGGTGGAGTAGAGCCCTGAG-3′
PPARγ	Forward 5′-CCACCAACTTCGGAATCAGCT-3′
	Reverse 5′-TTTGTGGATCCGGCAGTTAAGA-3′
LPL	Forward 5′-GTGACTCAGGAGTTCTGGGATAAC-3′
	Reverse 5′-GATTCACTTTTCTGGGACTGA-3′
GAPDH	Forward 5′-CCTACACCACCAACTGCTTA-3′
	Reverse 5′-GGCCATCCACAGTCTTCTGAG-3′
